# Evaluation of pulmonary complications in patients undergoing allogenic stem cell transplantation

**DOI:** 10.1186/s43168-020-00032-z

**Published:** 2020-10-01

**Authors:** Mohamed Zidan, Dalia Ahmed Nafea, Hadir Ahmed Said Okasha, Ahmed Farag Abouelnour, Heba Ahmed Eshmawey

**Affiliations:** 1 Chest Diseases Department, Faculty of Medicine, Alexandria University, Alexandria, Egypt; 2 Internal Medicine Department, Faculty of Medicine, Alexandria University, Alexandria, Egypt; 3 Microbiology Department, Faculty of Medicine, Alexandria University, Alexandria, Egypt

**Keywords:** Stem cells, Allogenic transplantation, Pulmonary complications

## Abstract

**Background:**

Mature blood cells can be differentiated from hematopoietic stem cells; thus, the latter can play a crucial role in maintaining defense against different microorganisms. Thus, hematopoietic stem cell transplantation is one of the most important lines of immunotherapy. Major systemic complications may occur post transplantation and could be fatal. Pulmonary complications include infectious and non-infectious complications. The aim of this study was to detect the pulmonary complications in allogeneic stem cell transplantation patients.

**Results:**

We studied 20 patients after transplantation of allogeneic stem cells with regular follow-up in outpatient clinic of hematology department of Alexandria Main University Hospital. All the studied patients were subjected to history taking, plain x-ray chest PA view, CT chest, complete blood count, serum creatinine, liver enzymes, and serum cytomegalovirus (CMV) detection by antibodies IgG and IgM. Regarding sputum sampling, 7 patients’ samples (35%) were obtained either spontaneously or by induction via hypertonic saline 3%. One patient (5%) had miniBAL done, while bronchoalveolar lavage using fiber optic bronchoscopy was done for 2 patients (10%). Samples could not be obtained from the remaining patients. Samples were analyzed for culture for bacteria, *Pneumocystis jiroveci* using immunofluorescence test, CMV PCR, fungal culture, and smear for acid fast bacilli (AFB). Among the examined patients, 2 patients (20%) had pulmonary bacterial infection including streptococcus and multidrug-resistant strain of *Klebsiella*, 3 patients (30%) had pulmonary candida infection, and one patient (10%) had positive result of pulmonary CMV of low count which was considered insignificant. None of our patients had positive results for pulmonary tuberculosis nor *Pneumocystis jiroveci*. Six patients (30%) had CMV in serum; 3 patients (15.8%) had manifested CMV reactivation. One patient (5%) of our patients had pulmonary graft versus host disease GVHD. One patient (5%) had died during our study course within 12 days post-transplantation due to ARDS followed by multiple organ failure.

**Conclusion:**

The prevalence of pulmonary infectious complications after allogenic stem cell transplantation was 50% of all studied patients, while 5% of the studied patients presented with non-infectious pulmonary complications.

## Background

It has been documented that the approved hematological malignancy therapies are effective in the control of the disease clinical and radiological deterioration. Nevertheless, no treatment can totally cure the patients from the original disease [1]. Hematopoietic stem cell transplantation (HSCT) takes place when a recipient’s stem cells are eradicated either by hematological malignancy, chemotherapy, or radiotherapy [2].

Stem cells are biological cells that have the ability to divide and differentiate into any cell type. Stem cell transplantation includes allogeneic stem cell transplantation in which the patient (recipient) receives stem cells from a donor, and autologous stem cell transplantation, in which stem cells are harvested from the patient himself and then reinfused again after completion of his treatment [3, 4].

Following allogeneic transplantation, the patient usually manifests systemic complications; complications are grouped into early or late [5, 6].

Early complications usually occur due to immunosuppressive state of the recipient; thus, most of them are due to infection, while late complications usually occur due to antigen-antibody reaction against the donor’s stem cells that end with graft versus host disease (GVHD) [7].

Acute graft versus host disease (GVHD) is characterized by fever, lethargy, rash, ARDS, liver dysfunction, and acute kidney injury and graft failure which could occur due to infection, recurrent disease, or poor count of donated stem cells [8].

Late complications include (a) idiopathic pneumonia syndrome (IPS), which has a rapid and progressive course that may lead to respiratory failure, (b) bronchiolitis obliterans syndrome (BOS) which is characterized by an obstructive pattern caused by air way inflammation, and (c) graft versus host disease (GVHD) which occurs when recipient’s immune system recognizes the donor’s transplanted cells as foreign body [9, 10].

Regarding pulmonary infectious complications, pneumococcus is considered the most common pathogen post-transplantation constituting 19% of all bacterial infections. While candida species is the most common fungal pathogen to be isolated from recipients’ post-transplantation as it causes 50% of fungal infections in these patients, cytomegalovirus (CMV) is the most common viral pathogen [11].

Roughly, 30% of HSCT patients will experience reactivation of latent CMV infection after allogeneic HSCT, which generally occurs within the late post-transplantation period and is linked with a mortality rate of 46% [12–14].

Skin GVHD is the most prevalent GVHD post-transplantation representing 80% followed by GIT (GVHD) with prevalence of 50%. However, GIT (GVHD) is considered to be the most fatal form. Furthermore, chronic GVHD (c-GVHD) is recorded in 60–80% of HSCT patients [15–17].

The aim of the present study was to detect the pulmonary complications in patients following allogeneic stem cell transplantation in Alexandria Main University Hospital. Minority of studies had concern with post-transplantation pulmonary complications. Our study is the first research in Alexandria Main University Hospital targeting the study of pulmonary complications after allogenic stem cell transplantation.

## Methods

This prospective observational study included 20 allogeneic HSCT patients. Till the date of data collection, this was the total number patients who did allogeneic HSCT in the unit. All the studied patients were above the age of 18 years. Patients were followed for 6 months in El Mowasah University Hospital. All the studied patients received prophylactic antibiotics, antifungal, and antiviral drugs as a part of HSCT protocol.

Written consent was taken before starting the study and also before bronchoscopy according to the guidelines of the ethics committee of Alexandria faculty of medicine.

During the follow-up period after transplantation, all the studied patients were subjected to:

Medical history, clinical examination, full laboratory investigations including complete blood count (CBC), serum creatinine, liver enzymes, and radiological examination including chest X-ray and CT chest.

Spirometry including forced expiratory volume in first second (FEV1), forced vital capacity (FVC), and FEV1/FVC ratio using (chestgraph HI-701).

### Sputum sampling

Samples were obtained either spontaneously in one patient who was able to produce sputum or by induction via hypertonic saline 3% in 6 patients. Mini-bronchial alveolar lavage (miniBAL) was obtained from the one studied intubated patient. MiniBAL is a process for obtaining blindly, not via bronchoscope, samples from the lower respiratory tract using a protected inner catheter. A small amount of saline solution was injected into the sterile catheter and then aspired to be analyzed.

Regarding patients who could not produce sputum, unfortunately, it was risky to expose the studied patients to an invasive procedure as bronchoscopy (for BAL) unless it was medically indicated as all the studied patients were immunocompromised receiving immunosuppressive drugs as a part of the procedure of transplantation. Only two studied patients had BAL done. One of them had middle lobar atelectasis, while the other patient had comprehensive bilateral pneumonia and retained secretions. Despite the utility of bronchoscopy in immunocompromised patients for diagnosis, about 50% are liable to complications. In addition, it may not change the decision of treatment or the outcome [18, 19].

Bronchoalveolar lavage using fiber optic bronchoscopy was done for two studied patients. The technique was done in endoscopy suite at the chest diseases department in Alexandria Main University hospitals under conscious sedation. Local anesthesia was given using a 10% local lidocaine spray introduced transnasally and/or transorally. Patients were sedated with incremental doses of IV midazolam. The procedure was done using Pentax video bronchoscopy (PENTAX EPK-15000/Tokyo, Japan) under continuous oxygen saturation and pulse monitoring.

Sputum, mini BAL, and BAL samples were subjected to bacterial culture and sensitivity via quantitative culture method, in which growth of an organism in a count > 10^6^ for sputum and > 10^4^ for BAL was considered significant [20].

Fungal culture was done on Sabouraud dextrose agar [21].

Smear for acid fast bacilli was examined using Ziehl Neelsen (ZN) stain [22].

*Pneumocystis jirovecii* detection was done via the immunofluorescence assay MONOFLUO KIT *P. jirovecii* (Bio-Rad, Canada) [23].

Quantitative CMV detection was done using real time PCR on collected pulmonary samples. DNA extraction from sputum, mini-Bal, and BAL samples was carried out using QIAamp DNA Mini Kit, followed by using the Artus CMV RG PCR Kit (QIAGEN) for quantitative detection of CMV DNA in the samples. The procedure was carried on Rotor-Gene 6000 Instrument. Results were expressed as copies/ml. Sample results were classified into low (less than 10^3^copy/ml), intermediate (between 10^4^ and 10^5^copy/ml), high (between 10^5^and 10^6^ copy/ml), and very high (more than 10^6^ copy/ml) according to number of copies/ml [24].

Serum samples were also tested for CMV-specific IgG and IgM antibodies by using enzyme-linked immunosorbent assay (ELISA) [25].

### Statistical analysis of the data

Data were fed to the computer and analyzed using the IBM SPSS software package version 20.0. **(**Armonk, NY: IBM Corp**)**. Chi-square test (Fisher or Monte Carlo) was used to compare between groups for categorical variables. Significance of the results was judged at the 5% level [26].

## Results

This study included 20 patients, 12 (60 %) of them were males and 8 (40%) were females. Regarding their original disease, four patients (20%) had T cell acute lymphocytic leukemia (T cell ALL), three patients (15%) had B cell acute lymphocytic leukemia (B cell ALL), and thirteen patients (65%) had acute myeloid leukemia (AML). One patient (5%) had died during the course of the study as shown in Table 1.

**Table 1:** Distribution of the studied cases according to different parameters (*n* = 20)

	No. (%)
**Gender (*n*** **= 20)**	
Male	12 (60%)
Female	8 (40%)
**Original disease (*n*** **= 20)**	
T cell ALL	4 (20%)
B cell ALL	3 (15%)
AML	13 (65%)
**Symptom (during study) (*n*** **= 20)**	
No	1 (5%)
**Yes**	**19 (95%)**
Cough	14 (70%)
Wheezes	11 (55%)
Fever	1 (5%)
Dyspnea	10 (50%)
Sputum	6 (30%)
Cyanosis	2 (10%)
Chest pain	2 (10%)
**Mortality (*n*** **= 20)**	
No	19 (95%)
Yes (ARDS)	1 (5%)

*ALL* acute lymphocytic leukemia, *AML* acute myeloid leukemia, *ARDS* acute respiratory distress syndrome, *GVHD* graft versus host disease

Table 1 also shows the patients who were symptomatic during the study. All patients showed symptoms overlap; most common concomitant symptoms were (cough and wheezes) in 75% of patients.

Spontaneous sputum was obtained from 1 patient (10%), 6 patients (60%) had induced sputum by hypertonic saline, fiber optic bronchoscopy was performed to 2 patients (20%), and one patient (10%) had miniBAL done.

Among the above 10 patients, nine patients (90%) had negative samples for CMV PCR using quantitative method, while only one patient (10% of patients’ samples) had a positive result for CMV in sputum of low count, which was considered insignificant. Regarding bacterial culture, 8 patients (80%) had negative results, one patient (10%) had *Streptococcus pneumoniae* infection, and one patient (10%) had sputum culture positive for multidrug-resistant *Klebsiella*. As for fungal culture, 7 patients (70%) had negative culture results, and 3 patients (30%) were positive for candida species. No mixed infections were detected.

Regarding ZN smear examination, all examined samples were found negative for acid fast bacilli; similarly, no samples were positive for *Pneumocystis jirovecii*. All patients were on prophylactic antimicrobials including (antibacterial, antiviral, and antifungal) during the period of study.

Thus, among the total number of studied 20 cases, 19/20 (95%) of patients had clinical signs while 12/20 (60%) of patients had radiological signs of lower respiratory tract infections; only ten patients had their sputum, miniBAL, or BAL samples examined; 5/10 of patients (50%) had positive bacterial or fungal culture. Thus, 5/10 (50%) of our patients had infectious pulmonary complications. Regarding noninfectious pulmonary complication, only one patient (5%) had pulmonary GVHD. Thus the total prevalence of pulmonary complications was 55%. Six of all studied patients had manifested pulmonary complications including infectious and noninfectious. The streptococcal pneumoniae infection occurred within the first month post transplantation, while the *Klebsiella* infection occurred on the third month post transplantation. Candida pulmonary infection occurred after 100 days period post transplantation, and the pulmonary GVHD (PGVHD) occurred after 200 days of transplantation.

As regards CMV reactivation, three of the patients’ serum results (15.8%) revealed elevated antibody titer indicating possible reactivation, one of which had seroconversion during study period, while 16 patients (84.2%) had negative serum results during follow-up. The seroconverted patient and one of the patients showing reactivation had suffered from extensive GVHD mainly mucocutaneous type.

Infectious extra pulmonary complications had occurred in two patients (10%) including *Klebsiella* bacteremia, and herpes zoster eruptions.

Among the studied patients, nine patients had manifested GVHD representing 45%. One patient (5%) had pulmonary GVHD, one had GIT GVHD, and seven patients had mucocutaneous GVHD including skin and corneal ulceration. One patient had complicated corneal ulceration that had ended by total eye blindness. Thus nine of our patients (45%) had suffered from non-infectious extra pulmonary complications.

**Fig. 1:**
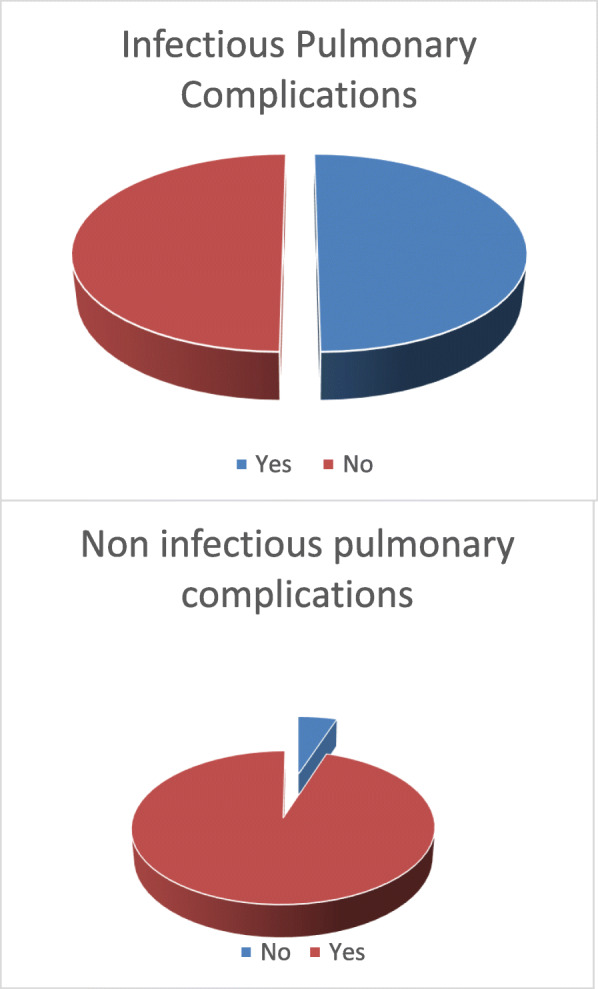
Prevalence of the pulmonary complications after allogenic stem cell transplantation

Table 2 and Fig. 1 show five patients (50%) had suffered infective pulmonary complications, while 1 patient (5%) developed non-infectious pulmonary complication in the form of pulmonary GVHD.

**Table 2:** Distribution of the studied cases according to pulmonary complications

	No. (%)
**Method to obtain sample(n = 10)**	
Induced sputum	6 (60.0)
F.O.B	2 (20.0)
Sputum	1 (10.0)
MiniBAL	1 (10.0)
**Bacterial culture (*n*** **= 10)**	
Negative	8 (80.0)
*Streptococcus pneumoniae*	1 (10.0)
*Klebsiella* with multidrug resistant	1 (10.0)
**CMV sputum (*n*** **= 10)**	
Negative	9 (90.0)
Weak positive	1 (10.0)
**AFB smear (*n*** **= 10)**	
Negative	10 (100.0)
**Fungal culture (*n*** **= 10)**	
Negative	7 (70.0)
Candida	3 (30.0)
**PJP (*n*** **= 10)**	
Negative	10 (90.0)
**CMV serum (*n*** **= 20)**	
Negative	14 (70%)
Positive	6 (30%)
**CMV reactivation (*n*** = 19)*	
No	16 (84.2%)
**Yes**	3 (15.8%)
**Infectious pulmonary complications(*n*** **= 10)**	
No	5 (50%)
**Yes**	5 (50%)
**Noninfectious pulmonary complications (*n*** **= 20)**	
No	19 (95%)
**Yes**	1 (5%)

*The presence of missed case regarding CMV reactivation category is explained by death of one patient within 12 days post-transplantation

*F.O.B* fiber optic bronchoscopy, *CMV* cytomegalovirus, *AFB* acid fast bacilli, *PJP Pneumocystis jiroveci* pneumonia

In regard to Table 3, eight patients (40%) had normal radiology, 3 patients (15%) had ground glass opacities, four patients (20%) had consolidative patches, two patients (10%) had interstitial fibrosis, one patient (5%) had cavitary lesion, one patient had infective fungal infection, two patients (10%) had scattered pulmonary nodules, and one patient (5%) had bronchiectatic changes.

**Table 3:** Distribution of the studied cases according to radiological signs (*n* = 20)

	No. (%)
**Radiology (X ray, CT) (*n*** **= 20**^**#**^**)**	
Normal	8 (40%)
Ground glass opacity	3 (15%)
Consolidation	4 (20%)
Interstitial fibrosis	2 (10%)
Atelectasis	2 (10%)
Cavitary lesion	1 (5%)
Pulmonary GVHD	1 (5%)
Infective fungal infection	1 (5%)
Scattered pul nodules	2 (10%)
Bronchiectasis	1 (5%)

Table 4 shows the interpretation of patients’ spirometry results.

**Table 4:** Interpretation of spirometry in studied patients (pulmonary function test) (*n* = 19^#^)

Spirometry (pulmonary function test)	No.	%
**Comment**		
Normal	15	78.9
Restrictive	3	15.8
Mixed obstructive and restrictive	1	5.3
**FVC percent predicted (%)**		
Min.–Max.	24.0–132.0	
Mean ± SD	88.63 ± 32.43	
Median	93.0	
**FEV1 percent predicted (%)**		
Min.–Max.	14.30 – 128.0	
Mean ± SD	85.46 ± 33.93	
Median	85.0	
**FEV1/FVC (%)**		
Min.–Max.	43.0–100.0	
Mean ± SD	78.74 ± 12.18	
Median	79.0	

*FVC* forced vital capacity, *FEV1* forced expiratory volume in first second

Nineteen patients had spirometry done. Fifteen of them (78.9%) had normal spirometry. Three patients (15.7%) had restrictive pattern, and one patient (5.3%) had mixed obstructive and restrictive pattern.

Table 5 shows the relation between mortality and infective parameters including bacterial culture and fungal culture. No significant relation was demonstrated between mortality and respiratory infective complications in our study.

**Table 5:** Relation between mortality and different parameters

Mortality		χ^2^	***p***
No (***n*** = 9)	Yes (***n*** = 1)		
**Bacterial culture (*n*** **= 10)**		1.810	^MC^*p* = 1.000
**Fungal culture (*n*** **= 10)**		0.476	^FE^*p* = 1.000

*χ*^*2*^ Chi square test, *MC* Monte Carlo, *FE* Fisher’s exact, *p p* value for comparing between the two categories

As shown in Table 6, no significant relation was found between CMV in serum reactivation and GVHD or extrapulmonary associated manifestations as *p* value was 1 in both of them.

**Table 6:** Correlation between CMV reactivation and different parameters

	CMV reactivation/ seroconversion				χ^2^	^**FE**^***p***
	No (***n*** = 16)		Yes (***n*** = 3)			
	No.	%	No.	%		
**GVHD (*n*** **= 19**^**#**^**)**						
No	8	50.0	1	33.3	0.281	1.000
Yes	8	50.0	2	66.7		
**Associated manifestation (*n*** **= 19)**						
No	6	37.5	1	33.3	0.019	1.000
Yes	10	62.5	2	66.7		

*χ*^*2*^ Chi square test, *FE* Fisher exact, *p p* value for comparing between the two categories, *GVHD* graft versus host disease, *CMV* cytomegalovirus

## Discussion

Pulmonary complications following stem cell transplantation are common and fatal. And they are classified into infectious and noninfectious complications [27]. The aim of our study was to assess pulmonary complications following allogeneic stem cell transplantation.

Bacterial pulmonary infections were detected in two patients in our study, the causative agents were *Streptococcus pneumoniae* and MDR *Klebsiella*, while three patients had candida as fungal infections; these infections were predisposed by effect of conditioning regimen on immune system. Long-term immunosuppression required for prevention or treatment of graft versus host disease (cGVHD) increases the susceptibility of transplanted patients particularly to infection with encapsulated organisms (*Neisseria meningitides*, *Streptococcus pneumonia*, and *Haemophilus influenzae*), fungi (*Candida* species, *Aspergillus* species, and *P. jirovecii*), and viruses (varicella zoster virus and cytomegalovirus CMV).

Streptococcal pneumoniae bacterial infection was found to be common in late post graft period, after 90 days from day 0 of transplantation followed by *Haemophilus* influenza, while gram-negative bacterial infection was found to be common in early post-engraftment period [28].

Our study was in agreement with Balletto et al. who found that streptococcal pneumoniae bacterial infection occurred in late post graft period, while *Klebsiella* infection occurred early post-engraftment [28].

As for fungal infection, our study has revealed that three patients were infected with candida representing about 33% of the studied samples; this occurred after 100 days post transplantation, while none of the samples was positive for *Pneumocystis jiroveci* nor *Aspergillus* infection, as all our patients were on prophylactic Sulfamethoxazole/trimethoprim and fluconazole.

Koldehoff and Zakrzewski et al. [29] found that the incidence rate of invasive fungal infection in hematopoietic stem cell transplant recipients was 10–26%, while Shi et al. had noted that mortality rate of patients with invasive fungal infection was 40–90% [30]. This is higher than the incidence of candida infection in our study which may be due to the difference in method of diagnosis as it was done by tissue lung biopsy and our small number of patients in our study. Furthermore, bronchoalveolar lavage was not done to patients who failed to obtain sputum sample, due to their immunocompromised state especially during early post engraftment period; however, fiber optic bronchoscopy was done essentially for 2 patients. One of them had middle lobar atelectasis, while the other patient had comprehensive bilateral pneumonia and retained secretions.

Risk considerations for invasive candidiasis include extended serious neutropenia, use of wide spectrum antibiotics, serious organ dysfunction, mucocutaneous injuries, and colonization with Candida species [31].

In our study, out of the 3 patients with invasive candida infection, one patient had radiological diagnosis of invasive fungal infection; this patient had positive candida fungal culture and was associated with extensive cutaneous GVHD.

Regarding noninfectious complications, GVHD was a common complication in our study as ten of our patients had manifested GVHD with its different types.

Taichung Veterans General Hospital as single institute retrospective study design had shown that acute GVHD (HR 2.98; 95% CI 1.27 to 6.95; *p* = 0.012) was significantly associated with CMV reactivation [32].

Similarly in our study, two patients had manifested CMV reactivation; one patient showed seroconversion during follow up; one of the reactivated and seroconverted patients was associated with GVHD manifestations.

Tuberculosis (TB) is a significant opportunistic infection of HSCT patients with an incidence of 2 to 40 times the general public [5]. However, the incidence has been found to be much higher in solid organ transplantation, especially renal transplantation, than HSCT. The negative tuberculosis results in our study could be explained by the fact that Egypt is currently classified by the WHO as a country of low incidence.

Graft versus host disease (GVHD) is considered a common and fatal complication that usually occurs after allogeneic stem cell transplantation in most of patients but with different degrees. In contrast to solid organ transplantation as kidney and liver transplantation, GVHD is more prevalent after HSCT. Chronic GvHD (c-GVHD), recorded in 60–80% of patients, is the most prevalent post-transplantation complication [17].

In our study, ten patients had manifested GVHD representing 50%. One of them had pulmonary GVHD, one had GIT GVHD, and seven patients had mucocutaneous GVHD including skin and corneal ulceration. One patient had complicated corneal ulceration that had ended with total eye blindness.

Administration of prophylactic immunosuppressive drugs had served to reduce the incidence of extensive GVHD in our study. The most common immunosuppressive agent that had been used was cyclosporine A.

In the center for International Blood and Marrow Transplant Research (CIBMTR), a research was done to evaluate the incidence and fates of grades II–IV acute GvHD during the three following periods 1999–2001, 2002–2005, and 2006–2012 [33].

There have been a number of interesting remarks. First, the acute GVHD severity appears over time to be decreasing. Secondly, in latest years, there have been fewer patients with simultaneous 3-organ implication (GIT/skin/liver) than in past years. Finally, in the tacrolimus immunosuppressant subgroup, the death risk and treatment-related mortality has been significantly reduced over time in patients with acute GVHD [34].

It has been reported in the International Bone Marrow Transplant Registry (IBMTR) that the incidence of acute GVHD may be as high as 60 to 80% in allogeneic HSCT recipients [15].

An 11-year retrospective study (between 1 January 2005 and 31 December 2015 performed in the University Hospital Wuerzburg) revealed that among allogeneic stem cell recipients, 60% had developed acute GVHD [8].

It was reported by Sorror et al. [35] a cumulative incidence of GVHD reaching 42% within 2 years in hematological malignant patients, while Ali et al. had reported a 29.9 % as an incidence [36].

Graft versus host disease (GVHD) is considered one of the causative pulmonary complications that may end with high mortality rate. In our study, one patient had died just after 12 days post-transplantation due to ARDS presented by extensive bilateral parenchymal consolidation.

In other studies about 35.2% of studied patients had died within first hundred days, and by the end of 2 years follow-up, the percentage had raised to 42.6%. The most common cause of delayed mortality was relapse of original disease and GVHD [37].

Discrepancy between our results and previous studies had worked on same topic may be explained by the small sample size and prophylactic use of antimicrobials and immunosuppressive drugs. In addition, the immunosuppressed status of the studied patients restrained the use of fiber optic bronchoscopy to obtain bronchoalveolar lavage.

## Limitation of the study

A small sample size and sputum study was done in only 50% of patients.

## Conclusion

Regarding pulmonary infectious complications post stem cell transplantation, the prevalence was 50% of all our studied patients, while 5% of our patients had manifested noninfectious pulmonary complications. Stem cell transplantation has been recently performed in Alexandria, and our study was restricted to patients who underwent allogeneic stem cell transplantation in El Mowasah University Hospital in Alexandria. Further studies including large number of patients are still needed in future.

## Data Availability

The datasets used and/or analyzed during the current study are available from the corresponding author on reasonable request.

## Abbreviations

CMV: Cytomegalovirus
PCJ: *Pneumocystis jiroveci*
AFB: Acid fast bacilli
GVHD: Graft versus host disease
PCR: Polymerase chain reaction
BAL: Bronchoalveolar lavage
ARDS: Acute respiratory distress syndrome
HSCT: Hematopoietic stem cell transplantation
IPS: Idiopathic pneumonia syndrome
BOS: Bronchiolitis obliterans syndrome
FEV1: Forced expiratory volume in first second
FVC: Forced vital capacity
PaCO_2_: Carbon dioxide partial pressure
PaO_2_: Oxygen partial pressure
HCO_3_: Bicarbonate level
SaO_2_%: Oxygen saturation
ALL: Acute lymphocytic leukemia
AML: Acute myeloid leukemia
ZN: Ziehl Neelsen
MDR: Multidrug resistance
TB: Tuberculosis
WHO: World health organization
CIBMTR: Center for International Blood and Marrow Transplant Research
IBMTR: International Bone Marrow Transplant Registry

## References

[1] Evers D, Zwaginga JJ, Tijmensen J, Middelburg RA, de Haas M, de Vooght KM (2017). Treatments for hematologic malignancies in contrast to those for solid cancers are associated with reduced red cell alloimmunization. Haematologica..

[2] Duarte RF, Labopin M, Bader P, Basak GW, Bonini C, Chabannon C (2019). Indications for haematopoietic stem cell transplantation for haematological diseases, solid tumours and immune disorders: current practice in Europe, 2019. Bone Marrow Transplant.

[3] Juric MK, Ghimire S, Ogonek J, Weissinger EM, Holler E, van Rood JJ (2016). Milestones of hematopoietic stem cell transplantation - from first human studies to current developments. Front Immunol.

[4] Biehl JK, Russell B (2009). Introduction to stem cell therapy. J Cardiovasc Nurs.

[5] Cho SY, Lee HJ, Lee DG (2018). Infectious complications after hematopoietic stem cell transplantation: current status and future perspectives in Korea. Korean J Intern Med.

[6] Pandey T, Maximin S, Bhargava P (2014). Imaging of complications from hematopoietic stem cell transplant. Indian J Radiol Imaging.

[7] Trajkovska I, Georgievski B, Cevreska L, Gacovski A, Hasan T, Nedeska-Minova N (2017). Early and late complications in patients with allogeneic transplantation of hematopoietic stem cell – case report. Open Access Maced J Med Sci.

[8] Hierlmeier S, Eyrich M, Wölfl M, Schlegel PG, Wiegering V (2018). Early and late complications following hematopoietic stem cell transplantation in pediatric patients - a retrospective analysis over 11 years. PLoS One.

[9] Ghimire S, Weber D, Mavin E, Wang XN, Dickinson AM, Holler E (2017). Pathophysiology of GvHD and other HSCT-related major complications. Front Immunol.

[10] Seo S, Renaud C, Kuypers JM, Chiu CY, Huang ML, Samayoa E (2015). Idiopathic pneumonia syndrome after hematopoietic cell transplantation: evidence of occult infectious etiologies. Blood..

[11] Sadon AA, El-Hagrasy R, Saraya M (2018). Pulmonary complications within the first year after bone marrow transplantation. Egyptian J Bronchol.

[12] Lachmann R, Loenenbach A, Waterboer T, Brenner N, Pawlita M, Michel A (2018). Cytomegalovirus (CMV) seroprevalence in the adult population of Germany. PLoS One.

[13] Azevedo LS, Pierrotti LC, Abdala E, Costa SF, Strabelli TM, Campos SV (2015). Cytomegalovirus infection in transplant recipients. Clinics (Sao Paulo).

[14] Mateos MK, O'Brien TA, Oswald C, Gabriel M, Ziegler DS, Cohn RJ (2013). Transplant-related mortality following allogeneic hematopoeitic stem cell transplantation for pediatric acute lymphoblastic leukemia: 25-year retrospective review. Pediatr Blood Cancer.

[15] Villarreal CD, Alanis JC, Pérez JC, Candiani JO (2016). Cutaneous graft-versus-host disease after hematopoietic stem cell transplant - a review. An Bras Dermatol.

[16] Naymagon S, Naymagon L, Wong SY, Ko HM, Renteria A, Levine J (2017). Acute graft-versus-host disease of the gut: considerations for the gastroenterologist. Nat Rev Gastroenterol Hepatol.

[17] Shokouhi S, Bray S, Bakhtiyari S, Sayehmiri K, Alimoghadam K, Ghavamzadeh A (2015). Effects of aGVHD and cGVHD on survival rate in patients with acute myeloid leukemia after allogeneic stem cell transplantation. Int J Hematol Oncol Stem Cell Res.

[18] Choo R, Naser NSH, Nadkarni NV, Anantham D (2019). Utility of bronchoalveolar lavage in the management of immunocompromised patients presenting with lung infiltrates. BMC Pul Med.

[19] Burger CD (2007). Utility of positive bronchoalveolar lavage in predicting respiratory failure after hematopoietic stem cell transplantation: a retrospective analysis. Transplant Proc.

[20] Scholte JB, van Dessel HA, Linssen CF, Bergmans DC, Savelkoul PH (2014). Roekaerts PM, et al Endotracheal aspirate and bronchoalveolar lavage fluid analysis: interchangeable diagnostic modalities in suspected ventilator-associated pneumonia?. J Clin Microbiol.

[21] Basu S, Bose C, Ojha N, Das N, Das J, Pal M (2015). Evolution of bacterial and fungal growth media. Bioinformation..

[22] Van Deun A, Hossain MA, Gumusboga M, Rieder HL (2008). Ziehl-Neelsen staining: theory and practice. Int J Tuberc Lung Dis.

[23] Fillaux J, Berry A (2013). Real-time PCR assay for the diagnosis of Pneumocystis jirovecii pneumonia. Methods Mol Biol.

[24] Schnepf N, Scieux C, Resche-Riggon M, Feghoul L, Xhaard A, Gallien S (2013). Fully automated quantification of cytomegalovirus (CMV) in whole blood with the new sensitive Abbott RealTime CMV assay in the era of the CMV international standard. J Clin Microbiol.

[25] Ikuta K, Koshizuka T, Kanno R, Inoue N, Kubo T, Koyano S (2019). Evaluation of the indirect and IgM-capture anti-human cytomegalovirus IgM ELISA methods as confirmed by cytomegalovirus IgG avidity. Microbiol Immunol.

[26] Asghar Ghasemi S (2012). Normality tests for statistical analysis: a guide for non statisticians. Int J Endocrinol Metab.

[27] Diab M, ZazaDitYafawi J, Soubani AO (2016). Major pulmonary complications after hematopoietic stem cell transplant. Exp Clin Transplant.

[28] Balletto E, Mikulska M (2015). Bacterial infections in hematopoietic stem cell transplant recipients. Mediterr J Hematol Infect Dis.

[29] Koldehoff M, Zakrzewski JL (2005). Modern management of respiratory failure due to pulmonary mycoses following allogenic hematopoietic stem cell transplantation. Am J Hematol.

[30] Shi JM, Pei XY, Luo Y, Tan YM, Tie RX, He JS (2015). Invasive fungal infection in allogeneic hematopoietic stem cell transplant recipients: single center experiences of 12 years. J Zhejiang Univ Sci B.

[31] Badiee P, Hashemizadeh Z (2014). Opportunistic invasive fungal infections: diagnosis & clinical management. Indian J Med Res.

[32] Lin HC, Han SM, Hwang WL, Chou CW, Chang KH, Shi ZY (2017). Cytomegalovirus infection and treatment in allogeneic hematopoietic stem cell transplantation: a retrospective study from a single institution in an endemic area. Turk J Haematol.

[33] Schoemans HM, Lee SJ, Ferrara JL, Wolff D, Levine JE, Schultz KR (2018). EBMT-NIH-CIBMTR Task Force position statement on standardized terminology & guidance for graft-versus-host disease assessment. Bone Marrow Transplant.

[34] Yanada M, Emi N, Naoe T, Sakamaki H, Takahashi S, Hirabayashi N (2004). Tacrolimus instead of cyclosporine used for prophylaxis against graft-versus-host disease improves outcome after hematopoietic stem cell transplantation from unrelated donors, but not from HLA-identical sibling donors: a nationwide survey conducted in Japan. Bone Marrow Transplant.

[35] Sorror ML, Martin PJ, Storb RF, Bhatia S, Maziarz RT, Pulsipher MA, et al (2014). Pre transplant comorbidities predict severity of acute graft versus host disease and subsequent mortality. Blood 124(2):287–95 10.1182/blood-2014-01-550566 PMC4093684 24797298

[36] Ali N, Adil SN, Shaikh MU, Masood N (2013). Frequency and outcome of graft versus host disease after stem cell transplantation: a six-year experience from a tertiary care center in Pakistan. ISRN Hematol.

[37] Mancuzo EV, Rezende NA (2011). Hematopoietic stem cell transplantation: pulmonary function tests and post-transplant mortality. J Bras Pneumol.

